# Virological failure reduced with HIV-serostatus disclosure, extra baseline weight and rising CD4 cells among HIV-positive adults in Northwestern Uganda

**DOI:** 10.1186/s12879-016-1952-x

**Published:** 2016-10-28

**Authors:** Jonathan Izudi, Sunday Alioni, Emmanuel Kerukadho, David Ndungutse

**Affiliations:** 1 Institute of Public Health and Management, International Health Sciences University, P.O. Box 7782, Kampala, Uganda; 2 Department of Anatomy, Uganda Society for Health Scientists, Makerere University College of Health Sciences, P.O. Box, 7072, Kampala, Uganda; 3 Arua District Local Government, Kampala, Uganda; 4 Baylor College of Medicine Children’s Foundation-Uganda, Box 72052, Kampala, Uganda; 5 Bugema University, School of Graduate Studies, P.O Box 6529, Kampala, Uganda

**Keywords:** Virological failure, CD4 cells, ART, HIV-serostatus disclosure, Northwestern Uganda

## Abstract

**Background:**

Little is known about the incidence of virological failure among Human Immunodeficiency virus (HIV) infected adults after Uganda transitioned from Zidovidine/Lamivudine/ Nevirapine (AZT/3TC/NVP) to Tenofovir/Lamivudine/Efavirenze (TDF/3 T/EFV) as a first-line anti-retroviral therapy (ART) in 2013. This was the first study in Uganda to investigate the incidence and predictors of virological failure among HIV-positive adults in Northwestern Uganda.

**Method:**

A retrospective cohort of 383 HIV-positive adults at Arua Teaching and Regional Referral Hospital HIV Clinic with at least six months of ART duration and five consecutive good adherence levels was used. Socio-demographic and clinical variables were analyzed with STATA version 12 at 5 % significance level. The Chi-squared, Fisher’s exact and Student’s t-tests were used for bivariate analysis. Cox Proportional Hazard Regression analysis was used for univariable and multivariate analysis, Kaplan-Meier for comparison of survival probability and the log-rank for testing survivorship probability. Hazard ratios (HR), 95 % confidence intervals (CI) and probability values were stated.

**Results:**

The average age of the cohort was 34.0 ± 11 years (Median: 32 years, Interquartile range (IQR): 25–31 years). 28 (7.3 %; 95 % Confidence Interval [CI]: 4.9-10.6) incident cases of virological failures and an incidence rate of 58 per 1000 person-years over risk time of 483 years was recorded. One-kilogram baseline body weight difference (41-kg and above) at ART initiation (Adjusted Hazard Ratio [aHR] = 0.86, 95 % CI:0.76-0.96, *P* = 0.008), one-CD4 cell increase (35 cells/ul and above) after ART initiation (aHR = 0.99, 95 % CI: 0.98-0.99, *P* < 0.001) and HIV-serostatus disclosure (aHR = 0.15, 95 % CI: 0.06-033, *P* < 0.001) reduced the hazard of virological failure.

**Conclusion:**

Virological failure is common among HIV-positive adults in Northwestern Uganda. It reduced with extra baseline weight, rising CD4 cell counts and HIV-serostatus disclosure.

## Background

Anti-retroviral therapy (ART) inhibits the replication of Human Immunodeficiency Virus (HIV) reflected by undetectable plasma HIV concentration (lower than 50 copies /ml) for as long as possible. Since the arrival of ART, many countries adopted ART regimes based on efficacy, durability, tolerability, ease of use, availability, continuity of supply and potential for future use. In 2011, Ministry of Health-Uganda ART guidelines recommended the use of Tenofovir-Lamivudine (TDF/3TC) with either Nevirapine (NVP) or Efavirenze (EFV) as the preferred first-line option and Zidovudine-Lamivudine (AZT/3TC) with NVP or EFV as a first-line alternative [1].

In June 2013, the 2011 ART guideline was revised to incorporate the World Health Organization (WHO) ART recommendations [2]. The revisions included the substitution of AZT for TDF in adult regimen and, AZT for ABC in children less than 10 years old or weighing less than 35 kg. This was critical because HIV resistance to TDF or ABC does not confer resistance to AZT and 3TCbut rather, preserve them for second-line ART use. Secondly, it increase the susceptibility of HIV resistant strains to protease inhibitors (PIs) and AZT/3TC [1, 3]. With this revisions, the preferred first line ART became TDF/3TC/EFV. TDF/3TC/EFV was preferred because of the relatively low toxicity profile compared to other ART combinations, potential for improved adherence due to low pill burden (once daily dosing), and low renal toxicity profile of 3 %, safe use in pregnancy and with anti-tuberculosis medications. Consequently, the first and second-line alternatives became AZT/3TC/NVP and AZT/3TC/EFV respectively [2].

However, ART is limited by drug interactions, drug resistances that reduce its potency, incidences of adverse reactions, need to ensure nearly perfect adherence of at least 95 %, and treatment failures [1]. Resultantly, viral load testing is nowadays preferred over clinical and immunological monitoring to determine HIV replication and to evaluate ART effect on HIV progression and aggressiveness. In addition, viral load testing is a good marker in monitoring a successful ART program particularly the effectiveness [1].

The change in ART policy led to enrollment of substantial number of HIV-positive adults on TDF/3TC/EFV. However, even with the known several challenges of ART, presently, there is paucity of information on the incidence and predictors of virological failure following the change in ART policy and subsequent implementation. This study therefore assessed the incidence, socio-demographic and clinical factors associated with virological failure among HIV-positive adults at Arua Teaching and Regional Referral Hospital HIV Clinic, North-Western Uganda.

## Method

**Fig. 1:**
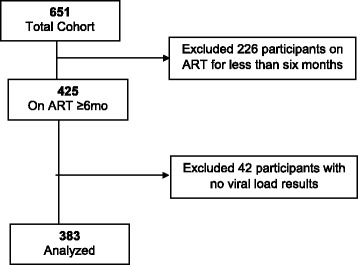
Study profile of HIV-positive adults at Arua Teaching and Regional Referral Hospital, Northwestern Uganda

This was a retrospective cohort study of HIV-positive adults enrolled on ART between June 2013 and December 2015. We analyzed data from 383 HIV-positive adults with five consecutive records of good ART adherence and ART use for at least six months (Fig. 1). Descriptive statistics of frequencies and percentages were computed and tabulated. In particular, numerical data was summarized into mean, standard deviation (SD), median and interquartile range (IQR).

The outcome variable was a composite measure of time to virological failure. Time was take as the duration from the date of ART initiation to the date of viral load assessment. Virological failure was defined as viral load greater than 5,000 copies/ml on two consecutive dry blood spot viral load measurements at least six months apart with adherence support; confirmed by plasma viral load of at least 1,000 copies/ml [2].

The independent variables were socio-demographic factors (sex, age, tribe, residence, marital status, occupation, education and religion) and clinical factors (CD4 before and after ART, body weight before and after ART, WHO clinical staging before and after ART, HIV serostatus and, functional status before and after ART). The incidence rate of virological failure was computed as the number of virological failures per 1000 person-years of observation.

Associations between the outcome variable and categorical independent variables were analyzed by Chi-squared test for larger cell counts (equals or more than five), or the Fisher’s exact test for smaller cell counts (less than five). Meanwhile, the Student’s t-test was used to analyze associations between numerical independent variables and outcome variable. Chi-squared, Fisher’s exact and Student’s *t*-test probability values (*P*-values) less than 0.2 together with clinically relevant independent variable(s) were taken significant for univariable Cox Proportional Hazard regression analysis. The results were stated in unadjusted hazard ratios (uHR) with, 95 % confidence intervals (95 % CI) and *P*-values.

*P*-values less than 5 % at univariable analysis were considered significant for multivariable Cox Proportional Hazard regression analysis and, results stated in adjusted hazard ratios (aHR), 95 % CI and *P*-values. Multivariable analysis controlled confounders and effect modifiers resulting into correct conclusions. The Kaplan-Meier curve was used to compare survival probabilities for significant variable(s) and differences in survivorship of virological failure tested by log-rank test. All HIV positive adults had written informed consent at enrolment into HIV care. The HIV clinic observe a written policy on patient rights: privacy, confidentiality of information, autonomy and respect among others. The Institutional Review Board of Arua Teaching and Regional Referral Hospital approved this study.

## Results

### General characteristics of respondents

651 HIV-positive persons were enrolled on ART during the study period but 268 (41.2 %) were excluded. Of the 268, 226 (84.3 %) were on ART for less than six months and 42 (15.7 %) had no viral load results (Fig. 1).

Of 383 respondents, 214 (55.9 %) were above 30 years of age, 238 (62.1 %) were females, 294 (76.8 %) were rural residents (stay 15 km away from Arua town), 234 (61.1 %) were married, 167 (43.6 %) were self-employed, 154 (40.2 %) ended at secondary level of education, 203 (53.0 %) were Catholics and 290 (75.7 %) were Lugbara ethnic tribe (Table 1). The average age of participants was 34 ± 11 years (Median: 32 years, IQR: 25–41 years). The mean baseline weight was 58.42 ± 8.01 kg (Median: 56.0, IQR: 53–62.0) but ranged from 41.0-87.5 kg. Similarly, the mean most recent CD4 count was 378 ± 140 cells/uL (Median: 362, IQR: 286–453) with a range of 35–981 cells/uL.

**Table 1:** Socio-demographic characteristics

Variable	Total (*N* = 383)
Age in years	
Below or equals 30	169 (44.1)
Above 30	214 (55.9)
Gender	
Male	145 (37.9)
Female	238 (62.1)
Residence	
Rural	294 (76.8)
Urban	89 (23.2)
Marital status	
Single	54 (14.1)
Married	234 (61.1)
Separated	71 (18.5)
One partner is dead	24 (6.3)
Occupation	
Formal employment	79 (20.6)
Self-employment	167 (43.6)
Not employed	137 (35.8)
Educational level	
None	41 (10.7)
Primary	95 (24.8)
Secondary	154 (40.2)
Tertiary	93 (24.3)
Religion	
Catholic	203 (53.0)
Moslem	46 (12.0)
Anglican	134 (35.0)
Tribe	
Lugbara	290 (75.7)
Ma’ di	14 (3.7)
Luo	79 (20.6)

### Incidence of virological failure among HIV-positive adults on first-line ART in Northwestern Uganda

The incidence rate of virological failure was 58 per 1000 person-years over risk-time of 483 years. The cumulative number of virological failure was 28 (7.3 %; 95 % CI: 4.9-10.6).

### Univariable analysis of factors associated with virological failure among HIV-positive adults on first-line ART in Northwestern Uganda

Compared to males, female HIV-infected adults had 55 % reduction in the hazard rate of virological failure (Unadjusted hazard ratio (uHR) = 0.45, 95 % CI: 0.21-0.95, *P* = 0.035). HIV-positive adults in World Health Organization (WHO) clinical stages III & IV had 40.0 % lower hazard rate of virological failure compared to those in WHO Clinical stages I & II (uHR = 0.60, 95 % CI: 0.08-4.40, *P* = 0.612).

The incidence of virological failure was higher among HIV-infected adults with baseline body weight less or equals to 56 kg compared to those weighing over 56 kg. A baseline body weight of over 56 kg was associated with reduced hazard rate of virological failure compared to a less or equivalent weight (uHR = 0.39, 95 % CI: 0.17-0.89, *P* = 0.025. In addition, a baseline one-Kilogram body weight difference: 41 kg to 42 kg and over was associated with 8 % reduced hazard rate of virological failure (uHR = 0.92, 95 % CI: 0.86-0.98, *P* = 0.008).

We dichotomized the baseline CD4 cell count using the median value of 250 cells/ul. Our analysis indicated that, HIV-infected adults with a baseline CD4 count greater than 250.0 cells/ul significantly had reduced hazard rate of virological failure compared to those that had a baseline CD4 cell count less than 250.0 cells/ul (uHR = 0.26, 95 % CI: 0.11-0.61, *P* = 0.002). Similarly, for every 1-additional CD4 cell count increase above the minimum value of 41 cells/ul, the hazard rate of virological failure significantly reduced by 1 % (uHR = 0.99, 95 % CI: 0.99-1.0, *P* = 0.005).

The most recent CD4 cell count was dichotomized using the median value of 362.0 cells/ul. Analysis showed that HIV-infected adults with the most recent CD4 cell count more than 362.0 cells/ul significantly had reduced hazard rate of virological failure compared to those with less or equal to 362.0 cells/ul (uHR =0.34, 95 % CI: 0.15-0.81, *P* = 0.015). Additionally, for every 1-CD4 cell count increase above 35.0 cells/ul, the hazard rate of virological failure significantly reduced (uHR = 0.99, 95 % CI: 0.99-1.00, *P* < 0.001).

HIV-positive adults with undisclosed HIV-serostatus had higher incidence rate of virological failure compared to those with disclosed HIV-serostatus. Disclosure of HIV sero-status was associated with 82.0 % lower hazard rate of virological failure (uHR = 0.18, 95 % CI: 0.08-0.37, *P* < 0.001).

### Multivariable analysis of factors associated with virological failure among HIV-positive adults on first-line ART in Northwestern Uganda

After adjusted analysis, three factors (baseline body weight in kilograms, most recent CD4 cell count after ART initiation and HIV-serostatus disclosure) were significantly associated virological failure (Table 2). HIV-positive adults with a baseline body weight less or equal 56 kg had similar hazard rates of virological failures compared to those that had and more than 56 kg (Adjusted hazard ratio (aHR) = 1.14, 95%CI: 0.31-4.14, *P* = 0.845). But for every 1-kg body weight increase above baseline body weight of 41- kg, the adjusted hazard rate of virological failure significantly reduced (aHR = 0.86, 95 % CI: 0.76-0.96, *P* = 0.008).

**Table 2:** Incidence rate per 1000 person years, unadjusted and adjusted hazard ratios of association between patient and clinical factors with virological failure

Variable	Total No. = 383	Plasma Viral load (>5, 000 copies/uL) No. = 28	Incidence rate per 1000 person-years	uHR (95 % CI)	*P*-value	aHR (95 % CI) Adjusted for all variables with *P* < 5 % at unadjusted level & WHO clinical stage	*P*-value
Age in years							
Below 30	169 (44.1)	12	57.2 (32.5-100.7)	1			
Above 30	214 (55.9)	16	58.7 (35.9-95.7)	0.98 (0.46-2.08)	0.947		
Residence							
Rural	294 (76.8)	25	67.3 (45.5-99.7)	1			
Urban	89 (23.2)	3	26.9 (8.7-83.5)	0.40 (0.12-1.33)	0.134		
Marital status							
Single	54 (14.1)	6	103.2 (46.4-229.8)	1			
Married	234 (61.1)	15	50.6 (30.5-83.8)	0.36 (0.14-0.95)	0.05		
Separated	71 (18.5)	6	62.8 (28.2-139.8)	0.43 (0.13-1.35)	0.148		
One partner is dead	24 (6.3)	1	31.0 (4.4-219.8)	0.20 (0.02-1.67)	0.136		
Occupation							
Formal employment	79 (20.6)	7	72.1 (34.4-151.3)	1			
Self-employment	167 (43.6)	11	52.0 (28.8-93.9)	0.64 (0.25-1.67)	0.362		
Not employed	137 (35.8)	10	57.4 (30.9-106.8)	0.73 (0.28-1.91)	0.519		
Educational level							
None	41 (10.7)	4	76.1 (28.6-202.8)	1			
Primary	95 (24.8)	4	32.3 (12.1-86.0)	0.40 (0.10-1.61)	0.199		
Secondary	154 (40.2)	12	63.1 (35.9-111.2)	0.86 (0.28-2.67)	0.795		
Tertiary	93 (24.3)	8	68.9 (34.5-137.8)	0.96 (0.29-3.19)	0.946		
Religion							
Catholic	203 (53.0)	14	53.9 (31.9-91.0)	1			
Moslem	46 (12.0)	2	38.4 (9.6-153.4)	0.78 (0.18-3.46)	0.749		
Anglican	134 (35.0)	12	70.3 (39.9-123.7)	1.28 (0.59-2.77)	0.529		
Tribe							
Lugbara	290 (75.7)	17	47.3 (29.4-76.1)	1	1		
Ma’ di	14 (3.7)	1	57.7 (8.1-409.8)	1.20 (0.16-9.04)	0.860		
Luo	79 (20.6)	10	94.2 (50.7-175.2)	1.82 (0.83-3.94)	0.134		
Sex							
Male	145 (37.9)	14 (9.7)	82.0 (48.2-138.4)	1		1	
Female	238 (62.1)	14 (5.9)	44.9 (26.6-75.8)	0.45 (0.21-0.95)	**0.035**	0.45 (0.19-1.03)	0.059
Baseline WHO staging				1		1	
I/II	359 (93.7)	21 (5.8)	59.6 (40.9-86.9)	0.60 (0.08-4.40)	0.612	0.60 (0.08-4.80)	0.632
III/IV	24 (6.3)	7 (29.2)	33.9 (4.8-240.4)				
Baseline weight/Kgs							
Less or equals 56 Kgs	194 (50.7)	20 (10.3)	82.0 (52.9-127.1)	1		1	
More than 56 Kgs	189 (49.4)	8 (4.2)	33.5 (16.8-67.0)	0.39 (0.17-0.89)	**0.025**	1.14 (0.31-4.14)	0.845
1-kg increase				0.92 (0.86-0.98)	**0.008**	0.86 (0.76-0.96)	**0.008**
Baseline CD4 cells/uL							
Less or equals 250 cells/uL	192 (50.1)	21 (10.9)	92.3 (60.2-141.6)	1		1	
More than 250 cells/uL	191 (49.9)	7 (3.7)	27.4 (13.1-57.6)	0.26 (0.11-0.61)	**0.002**	0.32 (0.08-1.20)	0.091
1-CD4 cell increase				0.99 (0.99-0.10)	**0.005**	1.01 (1.00-1.01)	0.133
Recent CD4 cells/uL							
Less or equals 362 cells/uL	194 (50.7)	21 (10.8)	84.9 (55.3-130.1)	1		1	
More than 362 cells/uL	189 (49.3)	7 (3.7)	29.8 (14.2-62.4)	0.36 (0.15-0.85)	**0.021**	5.02 (1.11-22.72)	0.056
1-CD4 cell increase/uL				0.99 (0.99-1.00)	**<0.001**	0.99 (0.98-0.99)	**<0.001**
HIV sero-status disclosure							
No	68 (17.8)	14 (20.6)	177.9 (105.4-300.4)	1		1	
Yes	315 (82.3)	14 (44.4)	34.7 (20.5-58.5)	0.18 (0.08-0.37)	**<0.001**	0.15 (0.06-0.33)	**<0.001**

*Note*

-Percentages calculated as row percentages (n/N)

-Level of significance was 5 %

-Bolded *P*-values are significant at 5 %

-*uHR* Unadjusted Hazard Ratio, *aHR* Adjusted Hazard Ratio, *CI* Confidence interval, *WHO* World Health Organization, *CD4* Cluster of Cell Differentiation-4

HIV-positive adults with most recent CD4 cell count (while on ART) increase of more than 362 cells/uL compared to those with a less or equivalent increase had over fivefold higher adjusted hazard rate of virological failure (aHR = 5.02, 95 % CI: 1.11-22.72, *P* = 0.056). In addition, every 1-CD4 cell count increase above 35.0 cells/ul led to a 1.0 % reduction in the adjusted hazard rate of virological failure (aHR = 0.99, 95 % CI: 0.98-0.99, *P* < 0.001).

**Fig. 2:**
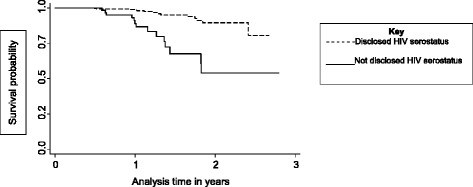
Kaplan-Meier survival probabilities between HIV-positive adults with and without sero-status disclosure, Arua Teaching and Regional Referral Hospital, Northwestern Uganda

Compared to none disclosure of HIV-serostatus, disclosure of HIV-serostatus (to a spouse, friend, relative or friend) was associated with 85.0 % reduction in adjusted hazard rate of virological failure (aHR = 0.15, 95 % CI: 0.06-0.33, *P* < 0.001). Actually, HIV-positive adults with disclosed HIV-serostatus had superior survival of virological failure than those with undisclosed HIV-serostatus (Fig. 2). This difference was highly statistically significant (Log-rank test, Chi-squared test value =27.3, 1° of freedom, *P* < 0.0001).

Similar hazard rates of virological failure (Table 2) were observed among female HIV-infected adults compared to males (aHR = 0.45, 95 % CI: 0.19-1.03, *P* = 0.059), among adults in baseline WHO clinical stage III /IV compared to WHO clinical stages I/II (aHR = 0.60, 95 % CI: 0.08-4.80, *P* = 0.632), between those with 1-CD4 cell count higher at ART initiation (aHR = 1.01, 95 % CI: 1.00-1.01, *P* = 0.133) and, between those with baseline CD4 cell counts less or equal to 250.0 cells/uL compared to those with baseline CD4 count more than 250.0 cells/uL (aHR = 0.32, 95 % CI: 0.08-1.20, *P* = 0.091).

## Discussion

Following revisions in the Uganda ART guidelines, this was the first study to investigate the incidence and factors associated with virological failure among HIV-positive adults at Arua Teaching and Regional Referral Hospital HIV clinic in Northwestern Uganda.

The cumulative incidence of virological failure was 7.3 % compared to 1.3 % among Ethiopian HIV-positive adults that received care at private health facilities [4]. The incidence rate of virological failure was 58 per 1000 person-years much higher than 27.9 per 1000 person-years among HIV-infected adults in Thailand [5]. In South Africa, a 19 % cumulative incidence of virological failure was reported in a study that investigated the relationship between virological failure and drug refill visits. However, the South African study defined virological failure by lower plasma viral load of at least 50 copies/ml after at least three months of ART [6]. Unlike the present study, the duration of ART was shorter to ensure significant viral load suppression and the plasma viral load threshold was low.

Our results indicated that HIV-serostatus disclosure reduced virological failure. This was expected. It is common acceptance that disclosure of HIV serostatus reduce HIV transmission risk and positively influence ART adherence. In Tanzania, disclosure of HIV serostatus led to provision of emotional and financial support to HIV-infected adults and improved adherence to ART [7]. Although HIV serostatus disclosure is very important, it must be well-handled to prevent loss of support from friends and family members [8]. Indeed in some quarters, HIV serostatus-related discrimination and divorce was reported [7].

The relationship between virologic failure and HIV serostatus disclosure is somewhat limiting. One study in Tanzania on HIV serostatus disclosure in a treatment cascade found no evidence in support of HIV serostatus disclosure and undetectable viral loads. Interestingly, nondisclosure of HIV serostatus led to lost linkage to HIV chronic care [9]. Unlike our findings, a study in Ethiopia reported reduction in the incidence of virological failure among HIV-infected adults with undisclosed HIV serostatus [4]. This was very surprising because HIV serostatus disclosure is known to enhance psychosocial support, to boost ART adherence and ultimately treatment outcomes. With disregard to these contradictions, current psychosocial support approaches must help HIV-positive persons to disclose their HIV-serostatus to meaningful gain its benefits like family support in long term chronic care. Mostly, psychosocial support is key in ART adherence support, stigma and discrimination reduction and, retention in HIV care.

Our study found an extra 1-CD4 cell count rise above 35 cells/mm^3^ after ART initiation reduced the hazard rate of virological failure. This result needs cautious interpretation. Published reports indicate rising CD4 counts are undermined by very low positive predictive values in detecting suppressed viral loads and vice-versa [10]. Secondly, CD4 cell counts are ineffective in predicting early virological failure than viral load tests [1, 3]. Also, CD4 cell counts strongly correlate with virological failure at individual level than at group level [11]. In Thailand, recent CD4 cell count increase of more than 50 cells/mm^3^ was associated with immunological failure but not virological failure among ART-naïve HIV-infected adults on Protease Inhibitor containing regimen [5].

In South Africa, no significant association was found between CD4 cell count and virological failure in a study that examined the association between virological failure and drug refill visits [6]. Current recommendations promote viral load monitoring but, it is expensive and not easily afforded by most health systems in Africa [1, 3]. In Uganda, coverage of viral load testing is still very low in both urban and rural settings but, efforts in increasing the coverage across the country through training of health workers in sample collection is evident. Our study thus re-emphasis the contribution of CD4 cell counts in predicting virological failure before overt clinical failure in resource limited settings. In the absence of viral load tests, CD4 cell counts may be useful in clinical patient management.

We found a 1-kg baseline body weight above 41 kg reduced the hazard rate of virological failure. Contrary to our findings, two studies revealed an increased risk of virological failure with higher baseline body weight [12, 13]. Unlike this study, these evidence was among patients on ART regimen containing Protease Inhibitors. In Ethiopia, a baseline body weight less than 50 kg was associated with reduced hazard rate of virological failure [4]. This study and the Ethiopian study confirmed that HIV-positive adults with baseline body weight less than 50 kg have increased hazard rate of virological failure. However, the Ethiopian study was conducted in a private healthcare setting whereas the present study was in public healthcare setting. Healthcare workers in both private and public health settings must routinely conduct nutritional assessments (weight, height and mid-upper arm circumference) at baseline and during follow-up visits to improve patient clinical care.

Studies in Malawi [14] and Tanzania [15] underscored the importance of nutritional assessment in ART care. In particular, the study in Malawi found an increase in body mass index (BMI) (measured by weight/kg per height/m squared) by less than 0.5 kg/m^2^ and MUAC by less than 0.5 cm after two weeks of ART strongly associated with high risk of death among HIV-positive adults [14]. The risk of death associated with reduced BMI while on ART was highest within the first three months [14]. In Tanzania, loss of any body weight was associated with death among HIV-positive adults on ART [15]. The loss of body weight persisted to predict high risk of death up to 1-year of ART [15]. The past studies [14, 15] and present study highlighted the importance of nutritional assessment, counseling and support in HIV care, treatment and support services.

We found no association between gender, age, baseline CD4 cell count and WHO clinical staging with virological failure. This is consistent with previous study in resource limited setting [11].

## Conclusion

This study highlighted the magnitude and predictors of virological failure among HIV-positive adults following the implementation of the revised ART guidelines in Uganda at Arua HIV Clinic, Northwestern Uganda. The incidence rate of virological failure was low at 58 per 1000 person-years. Rising CD4 cell counts after ART initiation (above 35 cells/ul), HIV-serostatus disclosure and baseline body weight above 41 kg were associated with reduced hazard rate of virological failure.

### Recommendation

In resource poor settings (where routine viral load tests are not feasible), rising CD4 cell counts while on ART may still be an alternative option for monitoring patient response to ART and predicting clinical outcomes. Secondly, HIV-positive persons aged 12 years and above must be supported in HIV-serostatus disclosure. This will enable them derive the benefits of psychosocial support. Finally, good nutritional status is critical for successful ART outcomes. Particularly, deliberate efforts in nutritional assessment, counseling and support are urgently needed in HIV care.

### Limitations

Like other studies, our study had flaws. First, the use of secondary data limited the number of variables that would be essential in making conclusions on virological failure. Secondly, there was lack of qualitative data on patient experiences to explain the observed outcomes. However, these limitations do not override the results as efforts were taken to explicitly explain and relate the findings to previous published studies. Importantly, this study was the first in Uganda to examine virological failure after the Ministry of Health adopted the WHO ART guidelines with a policy shift to TDF/3TC/EFV as the preferred first-line ART option. We have therefore set an important benchmark for policy makers, prospective researchers and program managers in improving clinical care of HIV-infected persons in Uganda.

## Abbreviations

3TC: Lamivudine
ABC: Abacavir
aHR: Adjusted hazard ratioodds ratio
AIDS: Acquired immunodeficiency syndrome
ART: Anti-retroviral therapy
AZT: Zidovudine
CD4: Cluster of cell differentiation four
EFV: Efavirenze
HIV: Human immunodeficiency virus
MUAC: Mid-upper arm circumference
NVP: Nevirapine
uHR: Unadjusted hazard ratio
WHO: World Health Organization
